# Identification of non-cardiomyocytes marker genes in patients with diabetes and cardiomyopathy through single-cell analysis

**DOI:** 10.1371/journal.pone.0351057

**Published:** 2026-06-05

**Authors:** Wenze Yu, Hanglie Chen, Lihua Shi, Guofang Gao, Haihua Wang

**Affiliations:** 1 Department of Medical Affairs, The First People’s Hospital of Xiaoshan District, Xiaoshan Affiliated Hospital of Wenzhou Medical University, Hangzhou, Zhejiang, China; 2 Department of Clinical Pharmacy, The First People’s Hospital of Xiaoshan District, Xiaoshan Affiliated Hospital of Wenzhou Medical University, Hangzhou, Zhejiang, China; 3 Department of Pharmacy, The First People’s Hospital of Xiaoshan District, Xiaoshan Affiliated Hospital of Wenzhou Medical University, Hangzhou, Zhejiang, China; 4 Department of Hospital Administration Office, The First People’s Hospital of Xiaoshan District, Xiaoshan Affiliated Hospital of Wenzhou Medical University, Hangzhou, Zhejiang, China; Jordan University of Science and Technology Faculty of Medicine, JORDAN

## Abstract

**Background:**

Diabetic cardiomyopathy (DCM) is a diabetes-related myocardial disorder causing fibrosis, hypertrophy, and progressive diastolic and systolic dysfunction. This study aims to explore how metabolic, inflammatory, and fibrotic mechanisms in non-cardiomyocytes drive DCM to reveal new therapeutic targets.

**Methods:**

Single-cell RNA sequencing (scRNA-seq) was performed to investigate the role of non-cardiomyocytes in DCM, enabling the identification of cell types, gene expression dynamics, and intercellular communication networks in patients with type 2 diabetes. The scRNA-seq data were obtained from the GEO to investigate cell-type-specific contributions and heterogeneity across tissues. Metabolic pathway scores were calculated using scMetabolism. Moreover, cell trajectory analysis and cellular communication studies were performed to examine shared and disease-specific cell populations in diabetes and cardiomyopathy. CCK-8, colony formation, Transwell migration and invasion assays were preformed to explore the function of PTPRC in HUVECs.

**Results:**

Using SingleR annotation, we identified eight distinct cell types, with NK cells and smooth muscle cells representing the shared cell populations across both diseases. Cell trajectory analysis revealed three distinct branches based on gene expression over pseudotime, and the top differentially expressed genes in each cell type clustering into six categories. Metabolic pathway analysis predicted that epithelial cells, macrophages, and neurons as the most metabolically active across multiple pathways, highlighting metabolic heterogeneity among patient samples. Additionally, four key signaling pathways associated with NK cells and smooth muscle cells were predicted to emphasize the divergence in gene expression across cell types. PTPRC is implicated in diabetes and cardiomyopathy and functions as a positive regulator of HUVEC viability, clonogenic growth, migration, and invasion.

**Conclusion:**

This study demonstrates significant heterogeneity among non-cardiomyocytes in patients with diabetes and cardiomyopathy, highlighting the need for targeted therapeutic interventions to address these differences.

## Introduction

Diabetes mellitus is a chronic metabolic disorder primarily characterized by persistent hyperglycemia, affecting over 500 million people worldwide-a number projected to reach 700 million by 2045 [1]. Prolonged hyperglycemia leads to serious complications that significantly impair both quality of life and life expectancy. Diabetes is primarily classified into type 1 diabetes mellitus (T1DM) and type 2 diabetes mellitus (T2DM) [2–4].

Diabetic cardiomyopathy (DCM) is a severe complication of diabetes, characterized by structural and functional myocardial abnormalities that occur in the absence of ischemic heart disease, hypertension, or other underlying cardiac conditions [5,6]. DCM is driven by metabolic dysregulation and microangiopathy, leading in widespread myocardial necrosis, which in turn results in structural abnormalities, myocardial fibrosis, and cardiomyocyte hypertrophy. Over time, these changes contribute to diastolic and/or systolic dysfunction of the left ventricle, progressing to heart failure, arrhythmia, cardiogenic shock, and in severe cases, sudden cardiac death [7,8]. The pathogenesis of DCM is intricate and involves a variety of factors, including disturbances in cardiomyocyte metabolism [9,10], myocardial interstitial fibrosis [11], oxidative stress [12–14], mitochondrial dysfunction [15,16], and inflammatory responses [17,18]. These mechanisms interact synergistically, ultimately driving progressive myocardial structural and functional abnormalities that may lead to heart failure.

In recent years, single-cell analysis techniques, particularly single-cell RNA sequencing (scRNA-seq), have significantly advanced the understanding of various diseases, including DCM [19–21]. These approaches have uncovered key drivers of intercellular communication in myofibrosis [22,23], mapped the cellular composition of the atherosclerotic aorta in mice [24], and characterized the phenotypic and metabolic heterogeneity of diabetes-associated atherogenesis [25]. Moreover, single-cell analysis has provided crucial insights into organ-specific changes associated with diabetic complications, offering a deeper perspective on the molecular mechanisms underlying DCM [26]. By enabling the exploration of cell-cell interactions among cardiomyocytes, fibroblasts, and endothelial cells, single-cell techniques have expanded our understanding of DCM pathophysiology. These findings lay a critical foundation for the identification of novel therapeutic targets, potentially guiding new intervention strategies for DCM.

In addition to cardiomyocytes, various non-cardiomyocytes are present in the heart and play a crucial role in the development and progression of DCM. These non-cardiomyocytes contribute to DCM through mechanisms such as myocardial fibrosis, endothelial dysfunction, inflammatory responses, and immune regulation [27–29]. Furthermore, the network of intercellular interactions in DCM is significantly altered, exacerbating myocardial injury and the progression of heart failure [30,31]. However, human DCM samples, particularly those suitable for scRNA-seq, remain scarce. To address this limitation, the present study integrated data from patients with type 2 diabetes and patients with cardiomyopathy to uncover cell types within samples, and investigated cellular and gene expression changes over pseudotime using cell trajectory analysis. Additionally, we explored intercellular communications among non-cardiomyocytes shared between diabetic and cardiomyopathy patients, identifying key signaling genes potentially involved in these interactions. This study aims to enhance our understanding of the contribution of non-cardiomyocyte to DCM and to identify potential targets for therapeutic intervention.

## Methods

### Data collection and preprocessing

The present study obtained scRNA-seq data related to diabetes and cardiomyopathy from the open access Gene Expression Omnibus (GEO) database using “diabetic cardiomyopathy” as the search query. The scRNA-seq data of human pancreatic islets of five patients with type 2 diabetes were obtained from GSE153855, based on the GPL16791 Illumina HiSeq 2500 (Homo sapiens) platform. In parallel, scRNA-seq data from eight patients with heart disease (coronary atherosclerotic heart disease and dilated cardiomyopathy) were retrieved from GSE121893, which was based on the GPL18573 Illumina NextSeq 500 (Homo sapiens) platform.

The Seurat package was used to create a Seurat object for each database. Data processing included filtering features detected in at least three cells and retaining cells with a minimum of 300 detected features. Cells with over 10% mitochondrial counts were removed using the PercentageFeatureSet function, and doublets were eliminated using the DoubletFinder function (PCs = 1:30, pN = 0.25). The two datasets were then merged for further analysis. Data integration was performed using SCTransform, which simultaneously carried out normalization, scaling, and identification of highly variable features, thereby ensuring consistency across datasets.

### Dimensional reduction and clustering

In this study, the 3,000 highly variable genes identified from SCTransform were subjected to principal component analysis (PCA) to reduce data dimensionality. Integration anchors were identified using FindIntegrationAnchors (dims = 1:30, reduction = “cca”), and the datasets were subsequently integrated with IntegrateData. For cell clustering visualization, several functions were utilized, including RunUMAP with dimensions set to 1:30, FindNeighbors with the same dimension parameter, and FindClusters.

### Cell annotation and marker genes

The “SingleR” package was employed for cell annotation, with the “HumanPrimaryCellAtlas” dataset from the celldex package serving as the reference. Given a reference dataset with known labels, cell type annotation for a test dataset is performed by assigning labels to test cells based on their similarity to the reference profiles. Subsequently, we sought to identify differentially expressed genes (DEGs) for each cell type using the “FindAllMarkers” function (test.use = “wilcox”, logfc.threshold = 0.25), and the proportion of each cell type was presented in a bar plot. Genes with a fold change greater than 0.25 and an adjusted p-value less than 0.05 were selected as differential marker genes for further analysis. The top three differentially expressed markers in each cell type were selected, with expression levels illustrated in a dot plot and heat map. Marker genes were extracted, and c2 hallmark pathways were explored through Gene Set Enrichment Analysis (minGSSize = 1, pvalueCutoff = 0.5).

### Cell trajectory analysis

Pseudotime analysis was performed using the Monocle2 package (version 2.24.0) to investigate cell developmental trajectories. A CellDataSet object was created using the newCellDataSet function. Data preprocessing included estimating size factors with estimateSizeFactors, estimating dispersions with estimateDispersions, and filtering genes with a minimum expression below 0.1 using detectGenes. Ordering genes were selected with setOrderingFilter. Dimensionality reduction was carried out using reduceDimension, and cells were ordered along the trajectory with orderCells to infer pseudotime. Visualization of cell trajectories were visualized with plot_cell_trajectory, grouping cells by Seurat clusters, division state, pseudotime, or cell types as specified by the grouping parameter.

The present study sought to identify genes exhibiting branch-dependent expression using branched expression analysis modeling (BEAM) to determine the cell fate decision of the top five marker genes of each cell type at a branch point. BEAM aims to identify genes with divergent expression between branches. The analysis was conducted with the following parameters: branch_point = 1, cores = 2, progenitor_method = “duplicate”. Results were visualized in a heatmap generated using the function plot_genes_branched_heatmap (branch_point = 1, num_clusters = 6, cores = 2). Furthermore, genes showing co-variation across pseudotime were clustered based on their expression levels and displayed in a heatmap (qval < 1e-4). To further explore pathway activity, gene set variation analysis (GSVA) was applied to estimate enrichment scores for each cluster.

### Metabolism score analysis

Metabolism score analysis was performed to assess single‑cell metabolic activities, given that DCM is a metabolic disease. Single-cell metabolism was quantified using the sc.metabolism.Seurat function (method = “VISION”, cores = 2, metabolism.type = “KEGG”). To visualize metabolic activity, the top 20 metabolic pathways were displayed using DotPlot.metabolism for each cell type and with a pheatmap for each sample.

### Cell-cell communications analysis

Intercellular communication was analyzed using the CellChat package. A CellChat object was created with the createCellChat function, using CellChatDB.human as the ligand–receptor interaction database. Overexpressed ligand–receptor pairs were identified with identifyOverExpressedGenes, and receptors/ligands were subsequently mapped onto the protein–protein interaction (PPI) network using projectData(). Communication probabilities were calculated with computeCommunProb (population.size = TRUE), and low-confidence interactions were removed using filterCommunication(min.cells = 3). Signaling pathway activities were inferred with computeCommunProbPathway and summarized using the aggregateNet() function.

Cell–cell communication networks were visualized using netVisual_circle, with pathway-specific ligand–receptor (L–R) interactions aggregated to generate comprehensive network representations. The contribution of individual L–R pairs within each signaling pathway was quantified using netAnalysis_contribution. Signaling gene expression patterns were displayed as violin plots generated by plotGeneExpression. Key network roles—including dominant senders, receivers, mediators, and influencers—were identified by computing centrality scores with netAnalysis_computeCentrality. Pathway signaling strength was visualized as heatmaps, highlighting outgoing and incoming signals across cell populations. Communication patterns were extracted using non-negative matrix factorization (NMF), and pattern stability was evaluated with the selectK function, which applies Cophenetic and Silhouette metrics to determine the optimal number of patterns. Major signaling interactions were then characterized using identifyCommunicationPatterns.

### Cell culture and transfection

Human umbilical vein endothelial cells (HUVECs) were obtained from the Cell Bank of the Chinese Academy of Sciences (Shanghai, China) and maintained in DMEM containing 10% fetal bovine serum (FBS) at 37°C. Short hairpin RNAs (shRNAs) targeting PTPRC were synthesized by GenePharma (Shanghai, China). shPTPRC-1: GCT GCA CAT CAA GGA GTA ATT; shPTPRC-2: CCT TTC CTA CAG ACC CAG TTT. PTPRC cDNA was cloned into the pcDNA3.1 expression vector. Cell transfection was carried out using Lipofectamine 3000 (Invitrogen, Carlsbad, USA).

### RT-PCR and Western blotting analysis

Total RNA from transfected HUVECs cells was extracted using TRIzol reagent in accordance with the manufacturer’s instructions. The mRNA was then reverse transcribed into cDNA, and quantitative real-time PCR (qRT-PCR) was subsequently carried out [32]. HUVECs were lysed in extraction buffer, and protein concentrations were determined using a BCA assay. Equivalent amounts of protein were separated by SDS–PAGE and transferred onto PVDF membranes. The membranes were then blocked with 5% milk and incubated with an antibody against PTPRC (1:1000, ab318154, Abcam), or GAPDH (1:5,000 dilution, ab8245, Abcam). After washing, the membranes were incubated with secondary antibodies, and protein bands were visualized by chemiluminescence [33]. The original, uncropped Western Blot images are provided in S1 Data.

### CCK-8 assay and colony formation assay

Cell proliferation of transfected HUVECs was evaluated using a CCK-8 assay in 96-well plates at 48 and 72 hours. After adding 10 µL of CCK-8 reagent and incubating for 2 hours at 37 °C, absorbance at 450 nm was measured to quantify cell viability. Transfected cells were plated in 6-well plates and maintained in complete medium for 14 days to permit colony formation. 4% paraformaldehyde was used to fix cells for 15 minutes, and stained with 0.1% crystal violet was used to stain cells for 30 minutes at room temperature. Then, cells were rinsed with PBS, and the colonies were subsequently imaged and counted.

### Transwell migration and invasion assays

Cell invasion and migration were evaluated using Transwell chambers with or without Matrigel coating. Cells in serum-free medium were added to the upper chamber, while medium containing 10% FBS was placed in the lower chamber and incubated at 37 °C for 24 hours. Non-migrated cells were removed, and the invaded cells on the lower membrane surface were fixed with 4% paraformaldehyde, stained with Giemsa, and then imaged and quantified under a microscope.

### Statistical analysis

All statistical analyses were performed using R programming language (version 4.1.3) software. Data from in vitro experiments were analyzed using paired Student’s t-tests and one-way analysis of variance (ANOVA) with Tukey post-test. p-values less than 0.05 were considered statistically significant.

## Results

### Dimensional reduction and clustering

**Fig 1:**
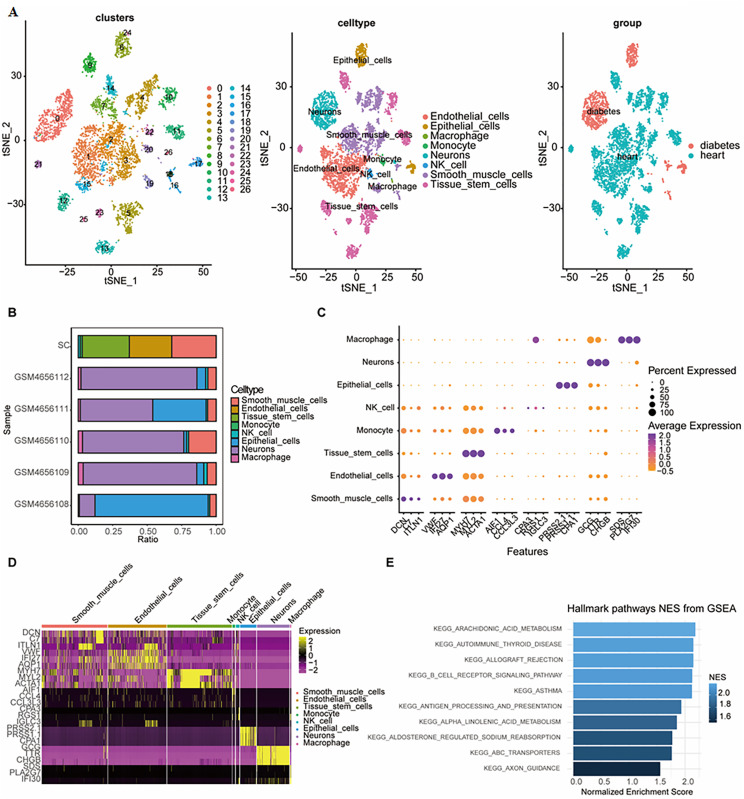
Overview of the diabetes and cardiomyopathy single-cell dataset across different aspects. **(A)** Genes were grouped into 27 clusters based on expression levels, followed by dimensionality reduction using t-SNE, visualized in a Dimplot. Cell annotation via SingleR classified the cells into eight cell types, represented by different colors. Most of the cells were derived from heart tissue. **(B)** The bar plot indicates the proportion of cell types across different samples. In heart tissues, smooth muscle cells, endothelial cells, and tissue stem cells were predominant, whereas islet tissues were primarily composed of neurons and epithelial cells. Smooth muscle cells and NK cells were the only common cell types between heart and islet tissues. **(C)** A dot plot presents the percentage and average expression level of the top 3 marker genes in each cell type, which indicates the heterogeneity of gene expression across different cell types. **(D)** The heatmap visualizes correlations among the top 3 marker genes in each cell type. **(E)** Among the top 10 enriched pathways of all marker genes, arachidonic acid metabolism exhibited the highest enrichment score.

After integrating the scRNA-seq data from five patients with diabetes and eight patients with cardiomyopathy, followed by quality control and filtering, we obtained a dataset of 28,829 cells, which were classified into 27 distinct cell types based on gene expression and visualized using t-distributed stochastic neighbor embedding (t-SNE) (Fig 1A). To further distinguish cell types, annotation was performed using the “SingleR” package, identifying a total of eight cell types. Most of these cells originated from heart tissues. To evaluate the accuracy of the SingleR‑based cell type annotations, we examined the expression patterns of well‑established canonical marker genes for each identified population. As shown in S1 Fig, each cell type exhibited specific and high expression of its corresponding markers. The full list of marker genes used for validation is provided in Table 1. These expression patterns were highly consistent with the SingleR annotations, with an exception of tissue stem cells, the overall validation supporting the biological plausibility of the assigned cell identities. A bar plot (Fig 1B) displays the distribution of cell types across samples, revealing that smooth muscle cells and natural killer (NK) cells were the predominant cell types in both diabetes and cardiomyopathy. However, significant heterogeneity was observed in cell-type proportions among different samples. The top three differentially expressed marker genes for each cell type were extracted and presented in a dot plot (Fig 1C). Notably, RGS1 was among the top three marker genes in NK cells and predicted to express high expression in macrophages. A heatmap illustrates the expression levels of the top three marker genes in each cell type (Fig 1D). Furthermore, GSEA was performed to identify hallmark pathways enriched across all marker genes, with the top 10 pathways presented in a bar plot (Fig 1E). These pathways included several related to acid metabolism and immunology system signaling or diseases.

**Table 1:** Reported canonical marker genes of each cell type.

Cell type	Marker genes
Tissue stem cells	PROM1, KIT, THY1, ENG
Smooth muscle cells	ACTA2, CNN1, CALD1
NK cell	NCAM1, NKG7, GNLY
Neurons	NEFL, NEFM, TUBB3, SNAP25
Monocyte	CD14, ITGAM, MS4A7
Macrophage	CD14, CD163, CD68
Epithelial cells	EPCAM, KRT8, KRT19
Endothelial cells	PECAM1, VWF, CD34

### Cell trajectory analysis

**Fig 2:**
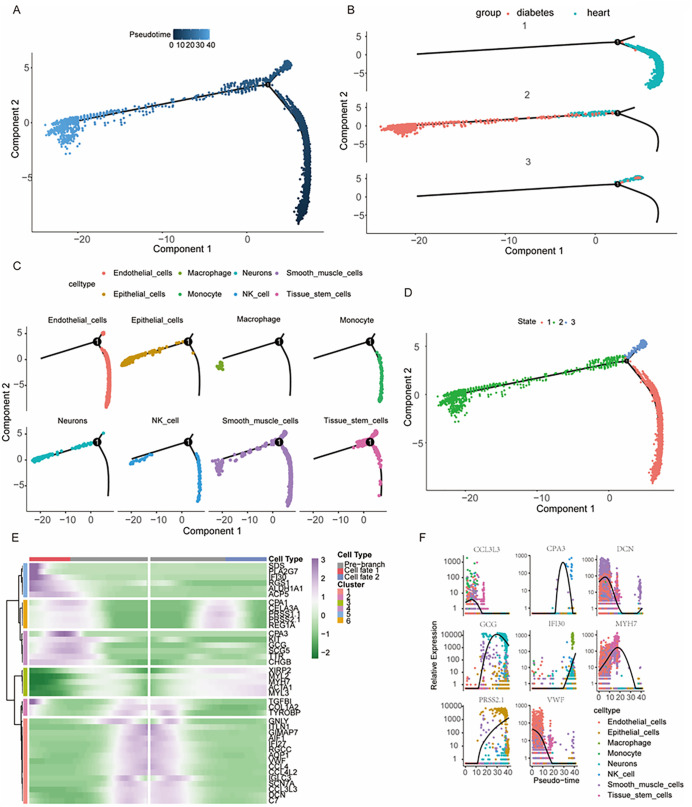
Cell trajectories in the diabetes and cardiomyopathy single-cell dataset and expression changes of marker genes over pseudotime. **(A)** Pseudotime trajectory plot, where each point represents a single cell. The color gradient indicates different stages of pseudotime progression. **(B)** The pseudotime trajectory was split in three groups, where the red group indicates the earliest cell division, which gradually develops into the blue and green groups. **(C)** The pseudotime trajectory was mapped for each cell type, with different colors representing branch points. **(D)** Trajectory plots were generated for each cell group, categorized by pseudotime states. **(E)** Genes responsible for distinguishing two distinct cell types from a progenitor cell were identified. The top five marker genes in each branch were selected, and six clusters were formed based on expression patterns over pseudotime, each representing a unique trajectory. **(F)** The highest-expressed gene in each cell type was plotted as a function of pseudotime, demonstrating expression level changes across different cell types in an ordered manner.

The pseudotime trajectory analysis delineated the developmental progression of cells within the dataset, identifying three-branches based on pseudotime (Fig 2A–2B). Cells followed a developmental trajectory from right to left, with a relatively short vertical axis. Scatter plots of each branch illustrated the disease-specific cell development trajectories, revealing that the origin of the trajectory predominantly consisted of heart tissue cells, whereas an increased proportion of islet cells was observed in branches 2 and 3 (Fig 2C). Furthermore, the trajectory was visualized in relation to cell types and pseudotime branches (Fig 2D). Notably, while smooth muscle cells and tissue stem cells were distributed across all three branches, other cell types followed distinct trajectories. For instance, macrophages were predicted to be appeared exclusively at the terminal point, suggesting they represent the most mature cells in the dataset, whereas monocytes were detected only in branch 1, indicating an earlier developmental stage.

The bifurcation of NK cells at both the initiation and termination points of the pseudotime curve was particularly notable (Fig 2E). Density plots further illustrated the expression distribution of cell types over pseudotime (S2A Fig). The top five marker genes for each cell type were extracted and clustered into six groups based on expression changes over pseudotime, with distinct bifurcation patterns observed at the branch point. For instance, cluster 6 displayed an initial decrease in expression, followed by an increase at the midpoint, and then a decrease again toward the end. In contrast, cluster 1 exhibited the opposite trend, with high expression levels that progressively declined through the final pseudotime stage.

A heatmap was used to visualize the expression patterns of the top genes in each cell type, regardless of the branch point (S2B Fig). These genes were grouped into six clusters, showing slight variations compared with the previous clustering results. To further investigate the functional differences among these clusters, GSVA was performed on each cluster (S2C Fig). This analysis revealed distinct pathway enrichment patterns across clusters, with the exception of one cluster that contained a single gene, highlighting the inherent heterogeneity in gene expression profiles. To further elucidate these findings, the most DEGs for each cell type were identified, and their expression changes over time were visualized in a scatter plot (Fig 2F).

### Quantify metabolism activity at single-cell level

**Fig 3:**
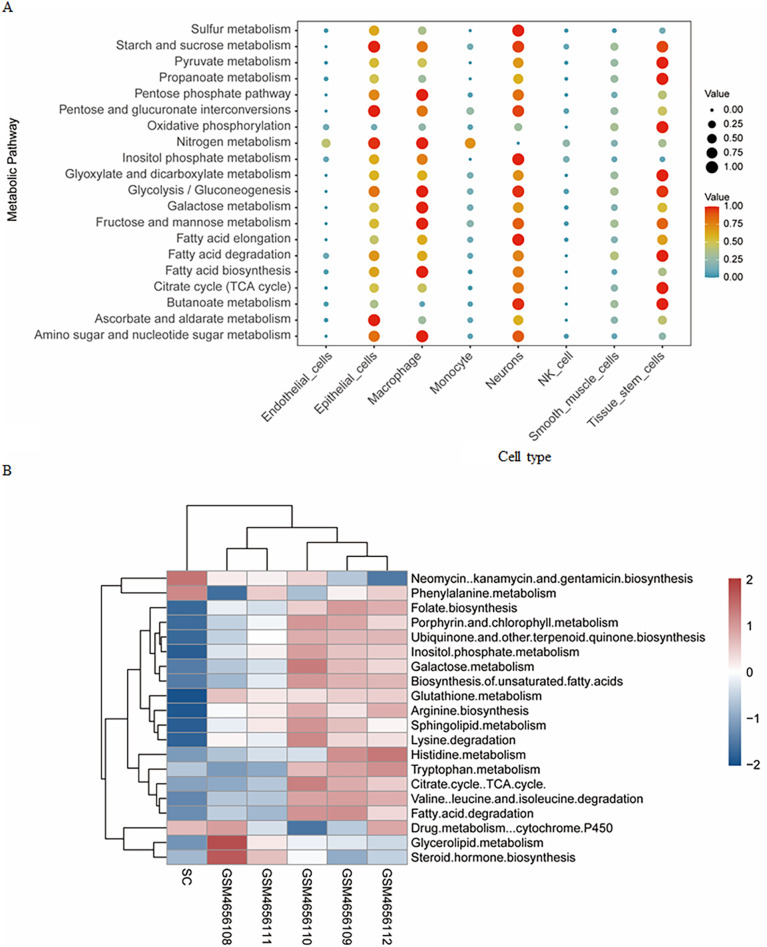
Single-cell metabolism activities across cell types and samples. **(A)** The dot plot represents the activity of the top 20 KEGG metabolic pathways in each cell type. High metabolic activity was observed in neurons, epithelial cells, macrophage, and tissue stem cells. **(B)** A heatmap depicting the top 20 metabolic pathways across samples based on normalized metabolism scores. Distinct metabolic patterns were observed between heart and islet tissues.

To quantify metabolic activity at the single-cell level, the scMetabolism tool was utilized to assess metabolic processes across different cell types, with the top 20 metabolic pathways presented in a dot plot (Fig 3A). Epithelial cells, macrophages, neurons, and tissue stem cells exhibited elevated metabolic scores, while endothelial cells and monocytes were predicted to show marked enrichment in the nitrogen metabolism pathway. A heatmap was used to present the mean metabolic scores of relevant pathways across individual samples (Fig 3B). In heart tissues, metabolic pathways were primarily enriched in neomycin, kanamycin, and gentamicin biosynthesis, phenylalanine metabolism, and drug metabolism cytochrome P450. However, the metabolic pathways in islet tissues displayed notable variations among samples. Samples on the right side of the heatmap exhibited a similar metabolic pattern, while GSM4656108 and GSM4656111 were particularly enriched in pathways related to neomycin, kanamycin, and gentamicin biosynthesis, as well as steroid hormone biosynthesis, with minor variations in other pathways.

### Cell-cell interaction analysis

**Fig 4:**
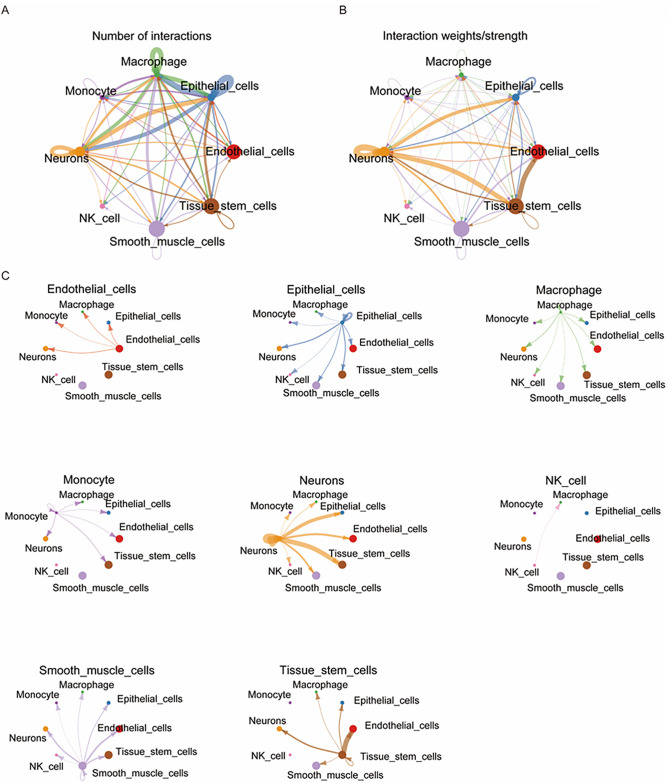
Cell-cell interaction network in integrated diabetes and cardiomyopathy data. **(A)** A circle plot illustrating the number of interactions among cell types. Notably, epithelial cells and neurons, as well as smooth muscle cell and macrophages, exhibited a substantial number of interactions. **(B)** A circle plot representing interaction strength among cell type. The strongest interactions were displayed between neurons and epithelial cells, neurons and tissue stem cells, and tissue stem cells and endothelial cells. **(C)** A circle plot displaying the number of outgoing interactions for each cell type. Interaction counts varied among cell types, with NK cells exclusively interacting with macrophages, suggesting immune cell-specific interactions.

Cell-cell interaction analysis was conducted using the Cellchat package to calculate the communication probabilities and the visualization of cell interactions. The number of interactions among different cell types is shown in a circular plot (Fig 4A), and the interaction weights of individual cells are presented in another circular plot (S3A–S3B Fig). A heatmap displays the number and strength of interactions among cell groups, revealing that the highest number of interactions occurred between macrophages and epithelial cells, epithelial cells and neurons, and smooth muscle cells and macrophages (S3C Fig). Additionally, a few interactions were observed from neurons, epithelial cells, and smooth muscle cells toward NK cells, whereas NK cells were predicted to send signals exclusively to macrophages. Another circle plot illustrated interaction strength, highlighting predominant communication from neurons to epithelial and tissue stem cells, as well as from tissue stem cells to endothelial cells. Notably, interaction strength involving NK cells was weaker than that that of other cell types (Fig 4B).

A panel of multiple circle plots demonstrated interactions sent from each cell type to the rest of cell populations (Fig 4C). Several cell types, including epithelial cells, macrophages, neurons, and smooth muscle cells, exhibited interactions with all other cell type. However, NK cells were predicted to interact exclusively with macrophages. Given that NK cells and smooth muscle cells were the only cell types common to both diabetes and cardiomyopathy, we extracted their metabolic pathway scores and re-analyzed them using scMetabolism. A comparison of the top 20 metabolic pathways, presented in a dot plot and heatmap, revealed differences in metabolic scores between the full dataset and the subset consisting solely of NK cells and smooth muscle cells (S4A–S4B Fig). These results indicate that NK cells and smooth muscle cells exhibit distinctly different metabolic pathways. In heart tissue, oxidative phosphorylation was a major contributor, whereas metabolic pathway heterogeneity persisted in islet tissues.

### Visualization of inferred signaling networks involving NK cells

**Fig 5:**
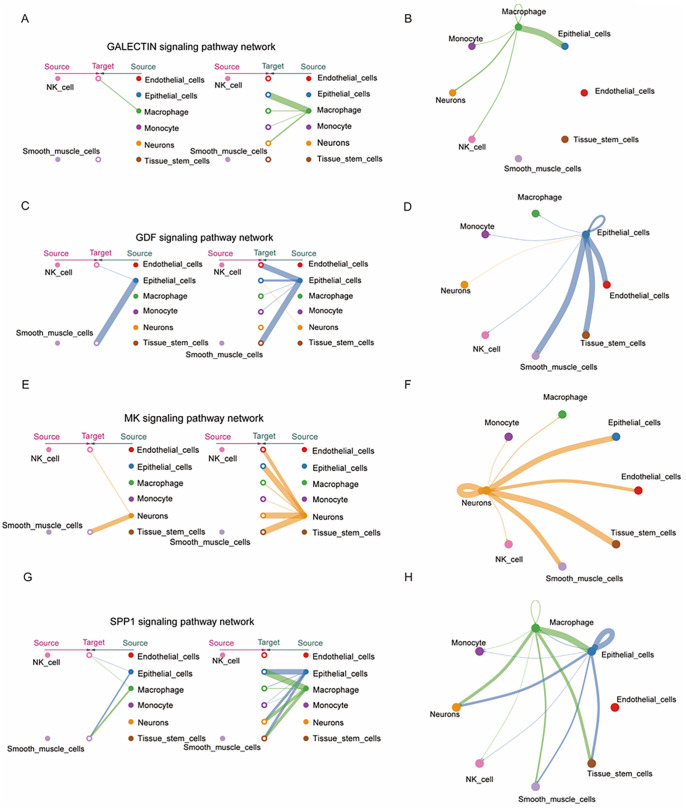
Visualization of inferred signaling networks involving NK cells. **(A-B)** Network centrality analysis indicated macrophages as the predominant source of GALECTIN ligands acting on NK cells. **(C-D)** The inferred GDF signaling network indicated that epithelial cells were primary source of GDF signals targeting NK and smooth muscle cells. **(E-F)** Neurons were identified as the dominant source of MK ligands acting on NK and smooth muscle cells. **(G-H)** Epithelial cells and macrophages emerged as primary sources of SPP1 signaling pathways affecting NK and smooth muscle cells.

Subsequently, we extracted signaling pathways that NK cells and smooth muscle cells targeted or received signals from other cell types. For instance, macrophages are predicted to send signals to NK cells but no significant ligand–receptor interactions were predicted between smooth muscle cells (Fig 5A–5B). In the GDF signaling pathway, both NK cells and smooth muscle cells were found to be target cells of epithelial cells, though the interaction strength between epithelial cells and smooth muscle cells was considerably stronger than between epithelial cells and NK cells (Fig 5C–5D). In the MK signaling pathway network, neurons are inferred to send signals to all other cell types, like the GDF signaling pathway. In contrast, the interaction between neurons and smooth muscle cells was significantly weaker than between neurons and NK cells (Fig 5E–5F). The SPP1 signaling pathway revealed that both epithelial cells and macrophages are predicted to interact with NK cells and smooth muscle cells (Fig 5G–5H).

### NK cell-related signaling networks and L-R pair contributions

**Fig 6:**
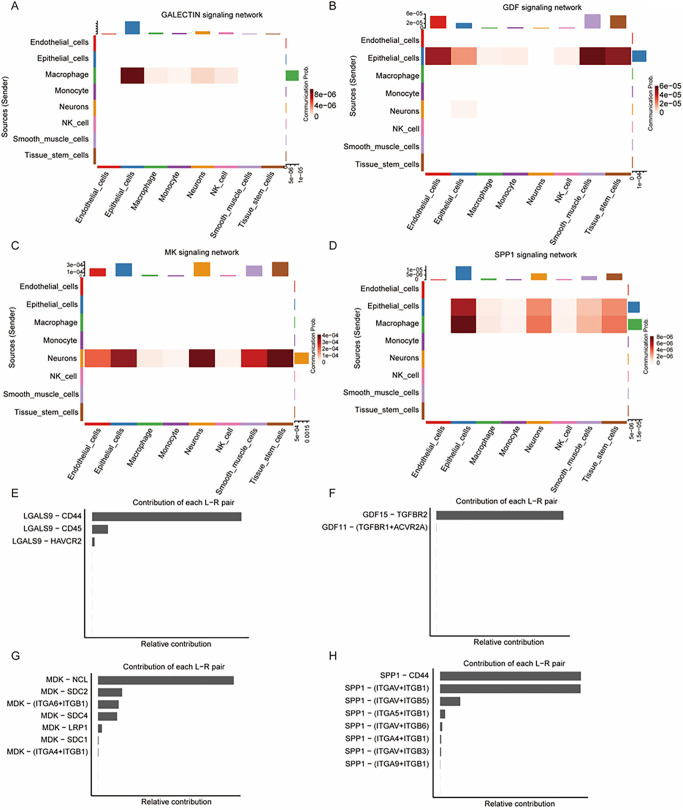
Heatmap of NK cell-related signaling networks and L-R pair contributions. **(A-D)** The selected signaling pathways (GALECTIN, GDF, MK and SPP1) targeting NK cells exhibiting varying interaction strengths with primary sender cells, although none were particularly pronounced. **(E)** The bar plot presents the contribution of each L-R pair in the GALECTIN pathway, highlighting LGALS9—CD33 as the predominant pair, followed by LGALS9-CD45 and LGALS9-HAVCR2. **(F)** In the GDF signaling pathway, the GDF15-TFGBR2 pair contributed most significantly, whereas the GDF11-(TGFBR1 + ACVR2A) pair had a minor contribution. **(G)** Multiple L-R pairs were involved in MK signaling, with MDK-NCL being the dominant pair. Other pairs, including MDK-SDC2, MDK-(ITGA6 + ITGB1), and MDK-SDC4, contributed similarly to the pathway. **(H)** In the SPP1 signaling pathway, the SPP1-CD44 and SPP1-(ITGAV+ITGB1) pairs contributed equally to the signaling network.

In the context of diabetes and cardiomyopathy, predicted signaling interactions among cell types were visualized based on cell color. Except for the GALECTIN signaling pathway, where no significant ligand–receptor interactions were inferred between macrophages and smooth muscle cells, the predicted interaction strength involving smooth muscle cells was higher than that of NK cells in the GDF, MK, and SPP1 signaling pathways (Fig 6A–6D). The contribution of each L-R pair within these pathways was computed and visualized in bar plots. In the GALECTIN pathway, three L-R pairs were predicted, including LGALS9-CD44, LGALS9-CD45, and LGALS9-HAVCR2, with LGALS9-CD44 contributing the most (Fig 6E). In the GDF signaling pathway, two inferred L-R pairs were observed: GDF15-TGFBR2 and GDF11-(TFGBR1 + ACVR2A), with GDF15-TGFBR2 showing the highest predicted contribution (Fig 6F). The MK and SPP1 pathways were inferred with more L-R pairs, with MDK-NCL in the MK pathway and SPP1-CD44 as well as SPP1-(ITGAV+ITGB1) in the SPP1 pathway demonstrating the strongest predicted contributions (Fig 6G–6H).

### Expression of signaling genes across cell types and heatmap of signaling strength

**Fig 7:**
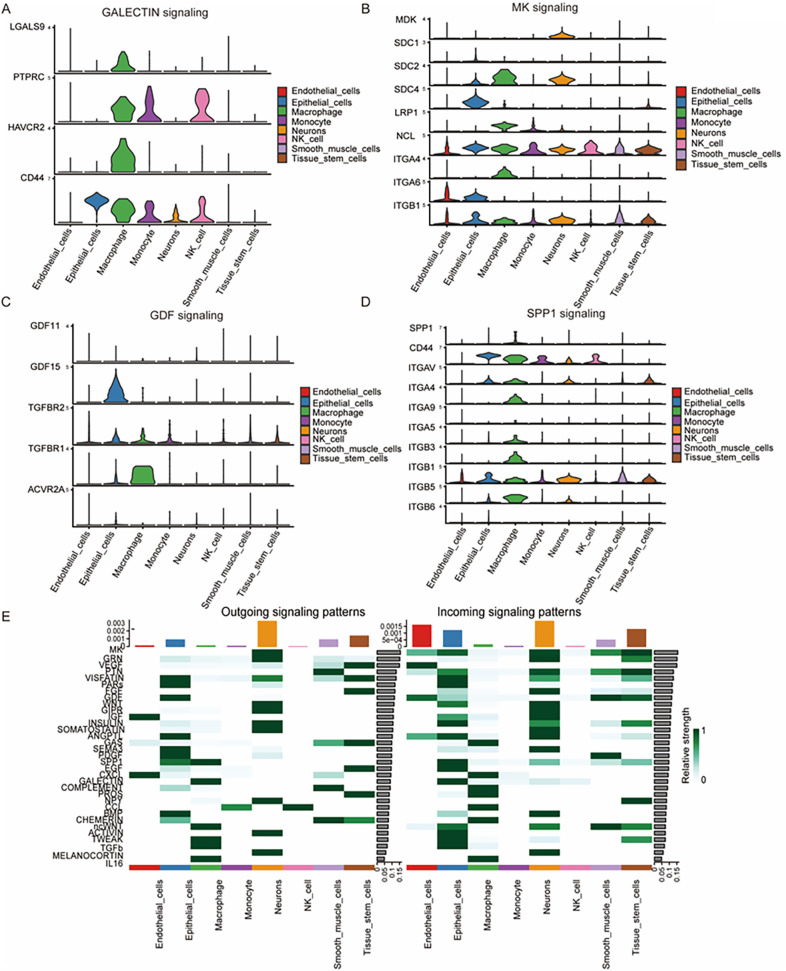
Expression of signaling genes across cell types and heatmap of signaling strength. **(A)** Among the four highly expressed genes in GALECTIN signaling, PTPRC and CD44 exhibited high expression levels in NK cells. **(B)** Nine genes were identified in the MK signaling pathway, with NCL being the only gene highly expressed in NK cells. **(C)** Genes involved in GDF signaling exhibited generally low expression levels, with GDF15 showing the highest expression in epithelial cells. **(D)** Genes in the SPP1 signaling pathway were highly expressed in macrophages, with CD44 also showing high expression in NK cell. **(E)** A heatmap displaying outgoing and incoming signaling patterns across all cell types. In terms of incoming signaling patterns, NK cells contributed weakly across all pathways. However, NK cells exhibited strong outgoing signaling in the CCI pathway, indicating a dominant signaling role in this network.

Furthermore, genes associated with the identified pathways were examined, and their expression patterns across cell types were visualized. In the GALECTIN signaling pathway, PTPRC and CD44 showed higher expression levels in NK cells compared to smooth muscle cells (Fig 7A). The MK signaling pathway involved nine genes, among which NCL exhibited high expression in both NK cells and smooth muscle cells, while ITGB1 was more prevalent in smooth muscle cells than in NK cells (Fig 7B). In the GDF pathway, TGFBR2 was relatively highly expressed in both NK cells and smooth muscle cells but remained at the lower end of the expression spectrum (Fig 7C). In the SPP1 signaling pathway, CD44, also implicated in the GALECTIN pathway, exhibited high expression level in NK cells, whereas ITGB1 was the only signaling gene highly expressed in smooth muscle cells (Fig 7D).

The predicted signaling strength of each pathway across all cell groups was visualized in a heatmap for both outgoing and incoming signaling (Fig 7E). NK cells were predicted to be primarily involved in the CCL pathway, whereas smooth muscle cells showed broader involvement across multiple outgoing pathways, including CHEMRIN, COMPLEMENT, and PTN. Conversely, NK cells exhibited lower prediction involvement in several incoming signaling pathways, including GALECTIN, SPP1, GDF, PTN and MK, which they shared with smooth muscle cells. A scatterplot illustrated the distribution of incoming and outgoing signaling, revealing that neurons had the most prominent involvement across all cell groups (S4C Fig). Furthermore, non-negative matrix factorization (NMF) was employed to identify key cell communication patterns.

The number of predicted signaling patterns was determined using two metrics, Cophenetic and Silhouette, with the first inflection point in the decreasing curve selected, which was three for outgoing signaling (S5A Fig). As a result, cells were categorized into three patterns, where NK cells and monocytes exhibited analogous patterns, being predominantly associated with patterns 1 and 2 (S5B Fig). Concurrently, communication patterns were identified within each signaling pathway. A similar methodology was applied to incoming signaling, revealing seven distinct patterns (S5C Fig). Among these, each cell group was clustered into a unique pattern except for NK cells and monocytes, which remained grouped together (S5D Fig). Comparing incoming and outgoing signaling patterns, the result indicated that outgoing patterns were more numerous and diverse. The dot plot elucidated that the sole outgoing communication pattern involving NK cells was associated with the CCL pathway (S5E-F Fig). In contrast, six outgoing signaling pathways were associated with smooth muscle cells, along with five associated incoming signaling pathways.

### PTPRC overexpression increases cell viability, colony formation, migration and invasion

**Fig 8:**
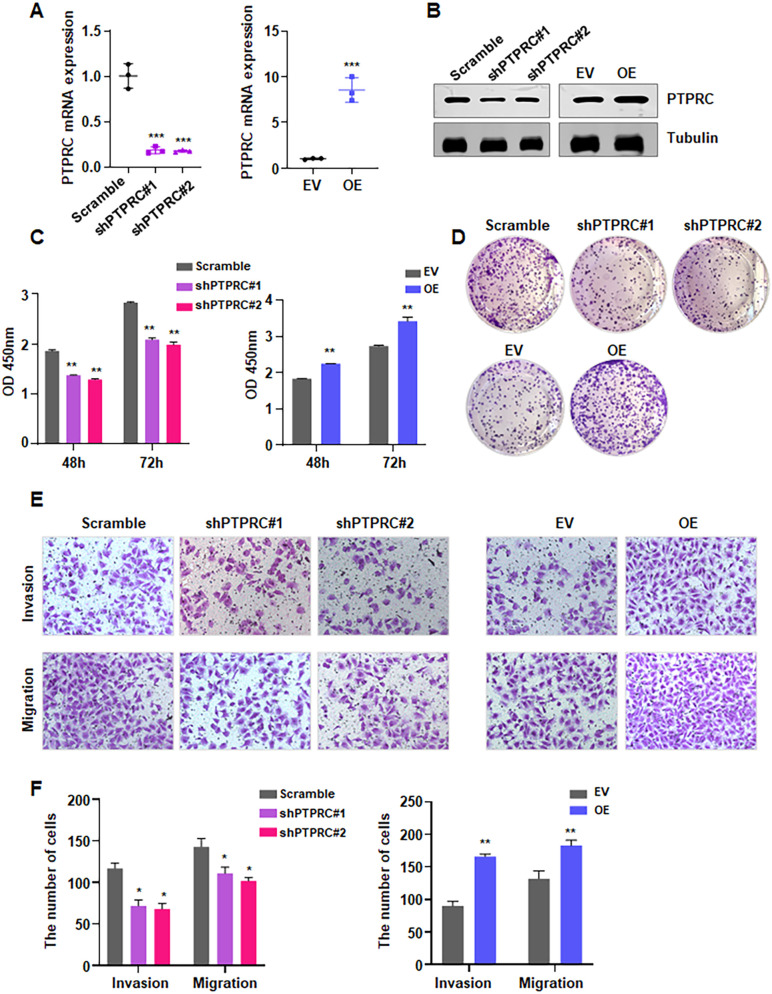
PTPRC overexpression increases cell viability, colony formation, migration and invasion. **(A)** RT-PCR analysis of PTPRC expression in HUVECs to assess transfection efficiency. EV, empty vector; OE, PTPRC overexpression; shPTPRC, PTPRC shRNA. **(B)** Western blot analysis of PTPRC protein levels in HUVECs following transfection. **(C)** CCK-8 assay assessing HUVEC viability after PTPRC modulation. **(D)** Colony formation assay evaluating clonogenic capacity of HUVECs after PTPRC modulation. **(E)** Transwell migration and invasion assays assessing the migratory and invasive abilities of HUVECs after PTPRC modulation. **(F)** Quantification of migrated and invaded cells shown in panel **E.** *p < 0.05; **p < 0.01; ***p < 0.001.

To investigate the role of PTPRC in regulating HUVEC viability, cells were transfected with either shPTPRC or PTPRC cDNA. Transfection efficiency was confirmed by RT-PCR and Western blotting. RT-PCR analysis showed that shPTPRC markedly decreased PTPRC mRNA expression, whereas PTPRC cDNA transfection significantly increased PTPRC mRNA levels in HUVECs (Fig 8A). Consistently, Western blotting demonstrated reduced PTPRC protein abundance following shPTPRC transfection and elevated PTPRC protein levels after PTPRC cDNA overexpression (Fig 8B). Functionally, CCK-8 assays revealed that PTPRC knockdown impaired, whereas PTPRC overexpression enhanced, HUVEC viability (Fig 8C). Similarly, colony formation assays showed decreased clonogenic growth in shPTPRC-transfected cells and increased colony formation in PTPRC-overexpressing cells (Fig 8D). Transwell assays further indicated that PTPRC silencing suppressed, while PTPRC overexpression promoted, HUVEC migration and invasion (Fig 8E–8F). Collectively, these findings indicate that PTPRC positively regulates cell viability, colony formation, migration, and invasion in HUVECs.

## Discussion

The most severe consequence of diabetes progression is an increased risk of cardiovascular disease (CVD) [34]. Individuals suffering from T2D have a two- to two-and-a-half times higher risk of cardiovascular complications in comparison with those without diabetes [35]. While the precise mechanisms of DCM remain unknown, further research into effective treatments is urgently needed. Current treatment strategies for DCM include non-pharmacological interventions such as dietary adjustment and regular physical activity [36–38] as well as medicine intervention, including beta-blockers, angiotensin-converting enzyme inhibitors (ACEi), and angiotensin receptor blockers (ARB) [39–42].

DCM is a metabolic disorder characterized by chronic hyperglycemia, which results in low-grade systemic inflammation [43]. Numerous studies have demonstrated that the inflammatory response plays a critical role in the onset and progression of DCM. The condition is exacerbated by NLRP3 inflammatory-mediated release of IL-1β and IL-18 [29,44], while interactions between RyR, Ca² ⁺ signaling, and the immune system may directly or indirectly affect cardiac function in diabetic patients [45–47]. Additionally, epigenetic modifications in macrophages influence their activation, inflammatory cytokine production, and tissue repair capacity, further exacerbating myocardial injury and fibrosis [48–50].

We acknowledge that integrating scRNA-seq data from pancreatic islets and heart tissues may introduce tissue-specific transcriptional biases. Although batch correction was applied, the observed shared cell populations (e.g., NK cells and smooth muscle cells) should be interpreted as exploratory and hypothesis-generating, rather than definitive evidence of cross-tissue disease mechanisms. Future studies using cardiac tissues from patients with DCM are needed to validate these findings. To date, direct studies on the relationship between DCM and NK cells remain limited. The predicted interactions between NK cells and macrophages across multiple signaling pathways suggest that these immune cells may play a role in DCM, indicating that NK cells could represent a relevant target for future mechanistic studies. Further exploration of the intricate molecular mechanisms underpinning the inflammatory process, as suggested by our computational analysis, may contribute to a deeper understanding of DCM pathogenesis and potentially inform the identification of new therapeutic targets. Metabolic profiling revealed a dichotomous pathway activation: NK cells exhibited enrichment in glycolysis and oxidative phosphorylation, while stromal smooth muscle cells demonstrated predominant engagement in fatty acid biosynthesis, amino sugar, and nucleotide sugar metabolism [51–53]. This metabolic compartmentalization fuels a vicious cycle in DCM progression, where stromal metabolic derangements drive fibrotic remodeling, concurrently compounded by NK cell metabolic failure that cripples immunosurveillance capacity.

Furthermore, analysis of the inferred cellular communication networks revealed potential ligand-receptor interactions among different cell types. This finding suggesting that tissue-specific communication patterns may exist. The present study identified smooth muscle cells as a hub in multiple inferred signaling networks, aligning with prior research suggesting that miR-21 regulates DCM by affecting vascular smooth muscle cell proliferation and apoptosis, cardiomyocyte growth and death, and cardiac fibroblast function [54]. A key limitation of this study is the lack of human cardiac tissue samples from patients with confirmed DCM, which necessitated the integration of diabetes and cardiomyopathy datasets. This approach has the potential to introduce biases due to tissue-of-origin effects and unresolved batch effects, potentially confounding the biological interpretation of shared cell populations. Consistent with recent single-cell studies of dilated cardiomyopathy and diabetic cardiac remodeling, we observed substantial heterogeneity among non-cardiomyocytes, particularly in fibroblasts and immune cells. He et al. identified M2 macrophage subpopulations, whereas our study extends these observations by revealing metabolic pathway enrichment in macrophages and predicting immune cell interactions [55]. In addition, Cohen et al. demonstrated fibroblast activation in a mouse model, and our human data are consistent with these findings while further identifying NK cells and smooth muscle cells as shared cell populations across diabetic and cardiomyopathic conditions [56].

PTPRC, also known as CD45, is a transmembrane protein tyrosine phosphatase expressed on virtually all nucleated hematopoietic cells and essential for antigen receptor–mediated signaling and activation in lymphocytes [57]. RNA-seq of filamin C (FLNC)-variant arrhythmogenic cardiomyopathy hearts identified 623 upregulated and 486 downregulated genes, implicating altered adhesion (JAM2, NEO1, VCAM1, PTPRC) and actin-cytoskeleton genes [58]. Using GEO myocardial fibrosis datasets and bioinformatic analyses, one study identified 635 DEGs enriched in immune and ECM remodeling pathways, highlighted hub genes including PTPRC [59]. Using two GEO mouse heart datasets, another study identified DEGs and enriched ECM, metabolic, and p53-related pathways in doxorubicin-induced cardiomyopathy [60,61], highlighting eight hub genes, including PTPRC, as potential mechanistic and therapeutic targets [62]. Galectin-3 is essential for MSC therapeutic effects in chronic Chagas cardiomyopathy, enabling MSC survival, migration, and suppression of cardiac inflammation, fibrosis, and CD45-associated immune activation [63]. Using combined transcriptomic and proteomic profiling of diabetic mouse kidneys, one group uncovered extensive immune-, autophagy-, inflammation-, and lipid metabolism–related alterations and hub genes including PTPRC, highlighting FN1, ICAM1, ANXA2, and APOA1 as potential therapeutic targets for diabetic nephropathy [64]. Using GEO transcriptomic data and network analyses, another group identified TLR4, ITGAM, ITGB2, PTPRC, and CSF1R as diabetes of the exocrine pancreas biomarkers [65]. Our study suggests that PTPRC is involved in patients with diabetes and cardiomyopathy and acts as a positive regulator of HUVEC viability, clonogenic growth, migration, and invasion. We recognize that HUVECs may not fully represent the immune cell-specific functions of PTPRC suggested by our scRNA-seq data. Therefore, the current functional experiments should be considered indirect and preliminary support for the bioinformatics findings. Further validation using primary cardiac endothelial cells or NK cells, as well as in vivo models, is warranted to confirm the role of PTPRC in DCM.

## Conclusion

In summary, this study integrated scRNA-seq data from patients with diabetes and cardiomyopathy to explore shared cell populations and cross-tissue heterogeneity. It is necessary to mention that reliance on public GEO datasets without detailed cohort matching or comprehensive clinical phenotyping limits both causal interpretation and translational relevance. The link between the identified cell–cell communication pathways and actual cardiac dysfunction remains largely inferential. Based on pseudotime analysis, genes were classified into six clusters according to their dynamic expression patterns. NK cells and smooth muscle cells were identified as the major cell populations shared between the two conditions. Several signaling pathways potentially involving these cell types were predicted, although the associated genes require further experimental validation. Collectively, these findings suggest that PTPRC may serve as a potential therapeutic target warranting further investigation in the context of diabetes and cardiomyopathy.

## Supporting information

S1 Data Raw Images. (PDF)

S1 Fig Validation of cell type annotations using canonical marker genes. (PDF)

S2 Fig Expression pattern of top marker genes over pseudotime. (PDF)

S3 Fig Metabolic pathway scores in NK cells across different samples. (PDF)

S4 Fig Signaling network pathways. (PDF)

S5 Fig Non-negative matrix factorization (NMF) identifies cellular communication patterns. (PDF)
