# Supplementary figures and images for: Identification of non-cardiomyocytes marker genes in patients with diabetes and cardiomyopathy through single-cell analysis
Source: PLoS One. 2026 Jun 5;21(6):e0351057. doi: 10.1371/journal.pone.0351057 (PMC13240930; doi:10.1371/journal.pone.0351057)

Fig 8B

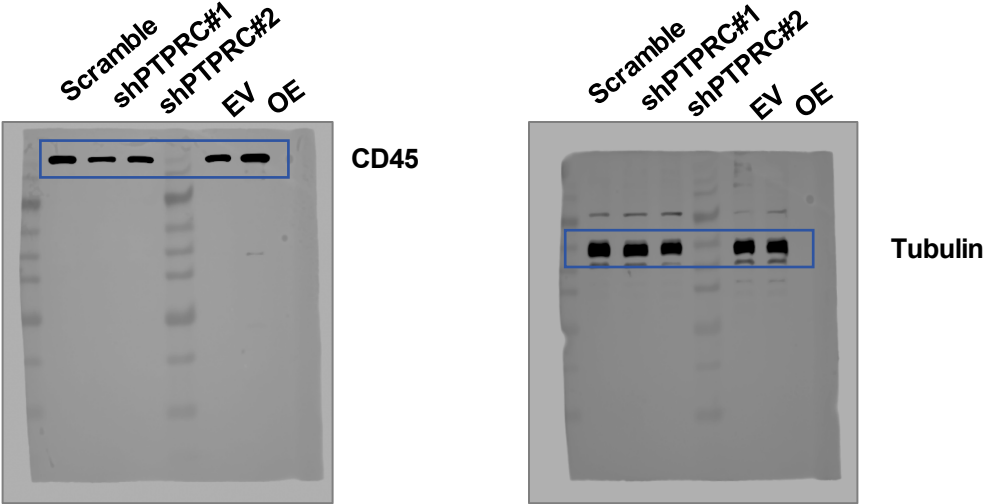

Supplement: S1 Data — (PDF) [file pone.0351057.s001.pdf]
