# Supplementary material for: Identification of non-cardiomyocytes marker genes in patients with diabetes and cardiomyopathy through single-cell analysis
Source: PLoS One. 2026 Jun 5;21(6):e0351057. doi: 10.1371/journal.pone.0351057 (PMC13240930; doi:10.1371/journal.pone.0351057)
Supplement: S1 Fig — (PDF) [file pone.0351057.s002.pdf]

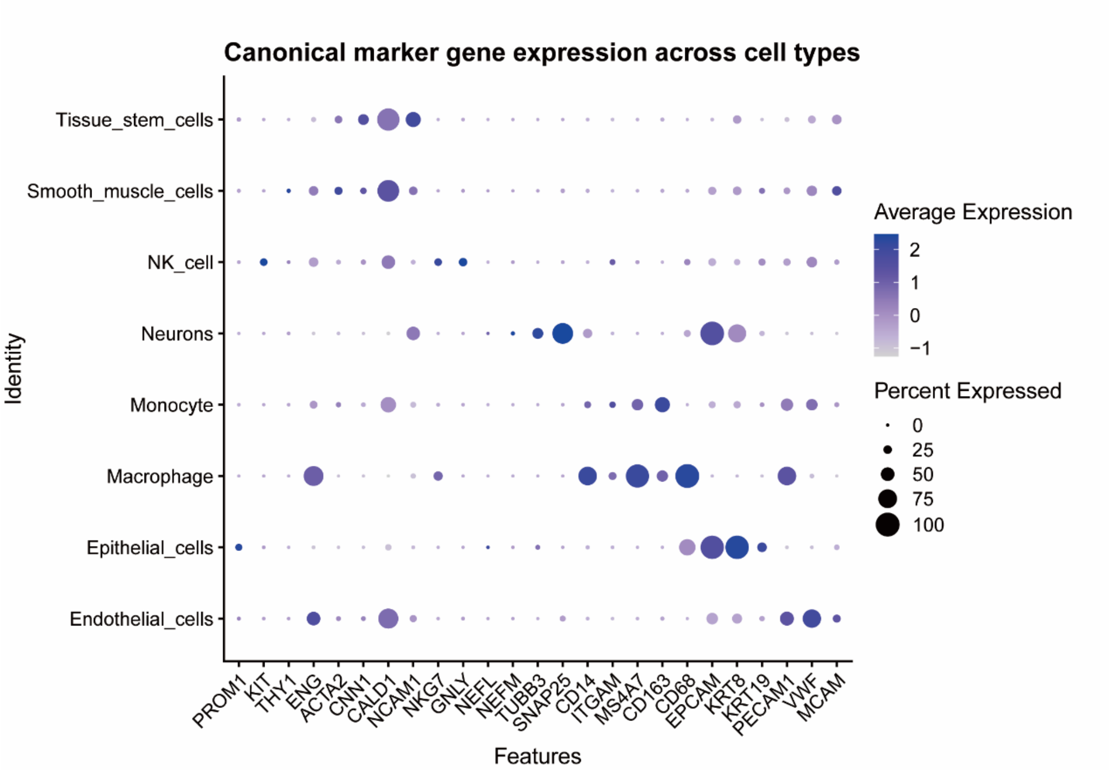

Supplementary Figure S1: Validation of cell type annotations using canonical marker genes. Dot plot showing the expression of selected canonical marker genes across the eight major cell types. Dot color indicates the average log-normalized expression level; dot size represents the proportion of cells expressing the marker.
