# Supplementary material for: Identification of non-cardiomyocytes marker genes in patients with diabetes and cardiomyopathy through single-cell analysis
Source: PLoS One. 2026 Jun 5;21(6):e0351057. doi: 10.1371/journal.pone.0351057 (PMC13240930; doi:10.1371/journal.pone.0351057)
Supplement: S3 Fig — (PDF) [file pone.0351057.s004.pdf]

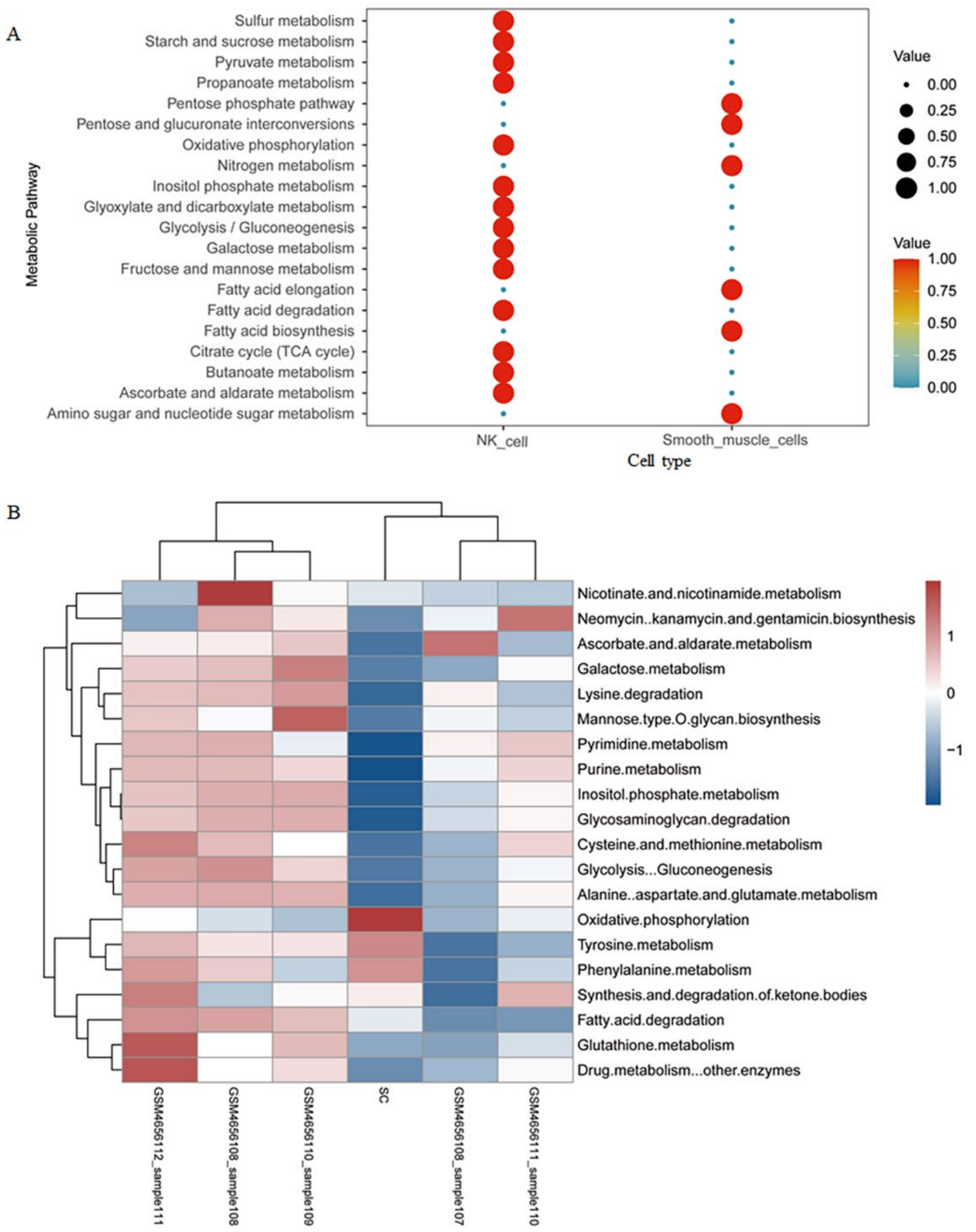

Supplementary Figure 3: Metabolic pathway scores in NK cells across different samples.

A) A dot plot displaying the metabolic scores of each pathway across samples. The results showed distinct metabolic score distributions among different tissues, with heart and islets tissues exhibiting distinct metabolic patterns.

B) A heatmap of top 20 metabolic pathways in NK cells across samples, revealing that only oxidative phosphorylation and mannose-type O-glycan biosynthesis were upregulated in heart tissues.
