# Supplementary material for: Identification of non-cardiomyocytes marker genes in patients with diabetes and cardiomyopathy through single-cell analysis
Source: PLoS One. 2026 Jun 5;21(6):e0351057. doi: 10.1371/journal.pone.0351057 (PMC13240930; doi:10.1371/journal.pone.0351057)
Supplement: S4 Fig — (PDF) [file pone.0351057.s005.pdf]

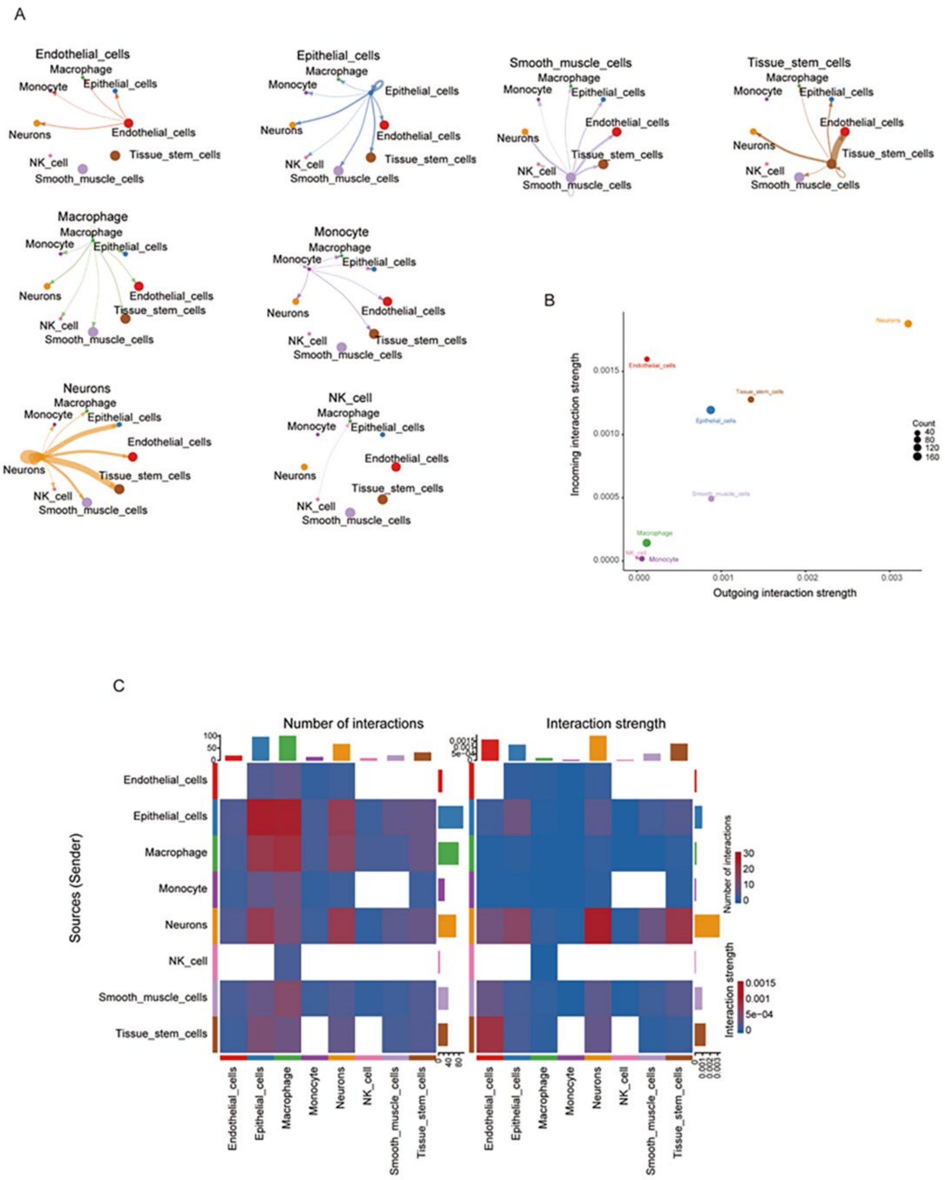

Supplementary Figure 4: Signaling network pathways.

A) A circular plot illustrating the interaction weights among different cell types in the dataset.

B) A scatterplot displaying the outgoing and incoming interaction strengths of various cell types, each represented by a distinct color.

C) A heatmap visualizing the number and strength of interactions for each cell type as a sender or receiver. The results indicate that epithelial cells, macrophage, and smooth muscle cells had relatively low interaction levels with NK cells.
