# Supplementary material for: Identification of non-cardiomyocytes marker genes in patients with diabetes and cardiomyopathy through single-cell analysis
Source: PLoS One. 2026 Jun 5;21(6):e0351057. doi: 10.1371/journal.pone.0351057 (PMC13240930; doi:10.1371/journal.pone.0351057)
Supplement: S5 Fig — (PDF) [file pone.0351057.s006.pdf]

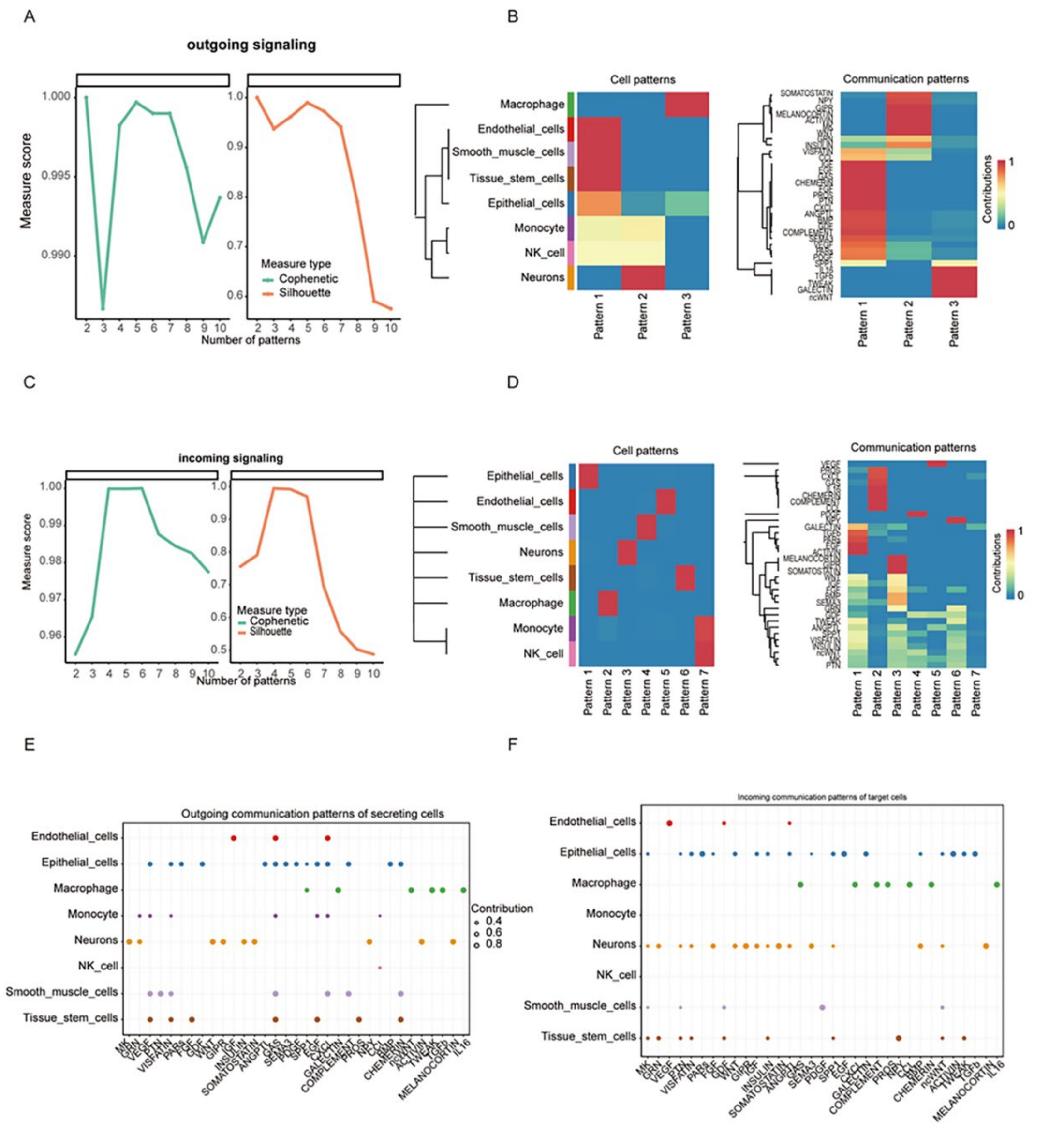

Supplementary Figure 5: Non-negative matrix factorization (NMF) identifies cellular communication patterns.

A) The number of communication patterns was determined using two NMF-based metrics, Cophenetic and Silhouette. The line plot indicated that outgoing signaling could be optimally grouped into three clusters.

B) Among the three identified outgoing signaling patterns, most cell types displayed different communication patterns, whereas monocyte and NK cells shared similar patterns.

C) The number of communication patterns for incoming signaling was also inferred

using Cophenetic and Silhouette metrics, with the line plot suggesting an optimal grouping into 7 clusters.

D) Similar to the outgoing signaling, among the 7 identified incoming signaling patterns, monocytes and NK cells displayed the similar communication pattern.

E-F) A dot plot illustrating the association between cell groups and their enriched signaling pathways in both outgoing and incoming signaling, based on contribution scores for each cell group.
