# Supplementary material for: Identification of non-cardiomyocytes marker genes in patients with diabetes and cardiomyopathy through single-cell analysis
Source: PLoS One. 2026 Jun 5;21(6):e0351057. doi: 10.1371/journal.pone.0351057 (PMC13240930; doi:10.1371/journal.pone.0351057)
Supplement: S2 Fig — (PDF) [file pone.0351057.s003.pdf]

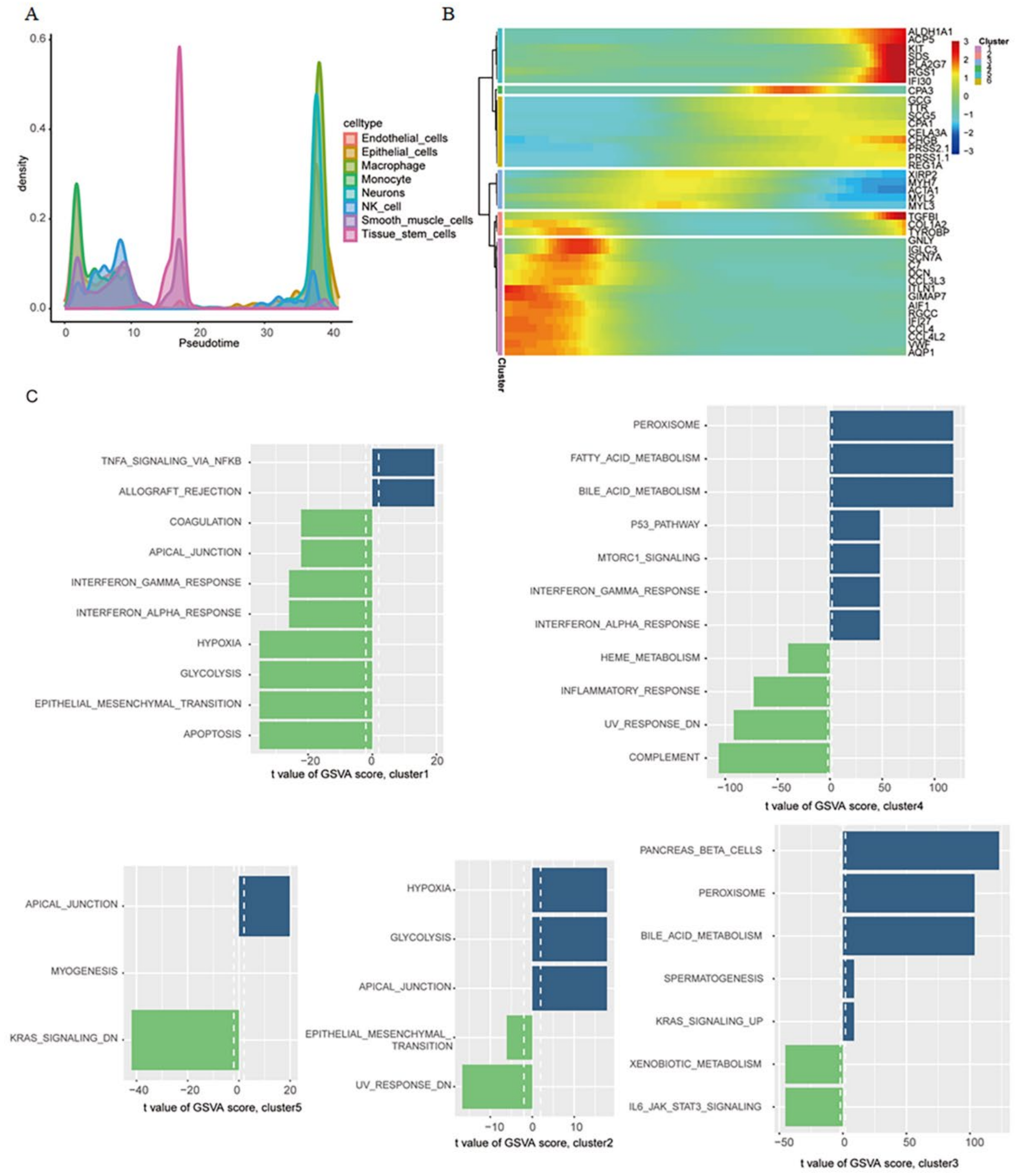

Supplementary Figure 2: Expression pattern of top marker genes over pseudotime.

A) Density distribution of cell types over pseudotime, revealing that tissue stem cells were clustered in the middle phase, while macrophage and neurons were predominant at the final stage.

B) The top 5 marker genes from each cell type were clustered into six groups based on their co-expression patterns across pseudotime.

C) Gene set variation analysis (GSVA) was conducted for each cluster, except for the cluster containing only one gene, to explore enriched pathways.
